# Efficacy of non-pharmacological interventions for individuals with amyotrophic lateral sclerosis: systematic review and network meta-analysis of randomized control trials

**DOI:** 10.1038/s41598-024-62213-w

**Published:** 2024-05-18

**Authors:** Zhao Li, Hyunsik Kang

**Affiliations:** College of Sport Science, Sungkyunkwan University, 2066 Seoburo, Jangan-gu, Suwon, Republic of Korea

**Keywords:** Amyotrophic lateral sclerosis, Non-pharmacological intervention, Network meta-analysis, Diseases, Health care, Medical research, Neurology

## Abstract

This network meta-analysis (NMA) aimed to compare the efficacy of five non-pharmacological interventions, including exercise intervention (EI), nutritional intervention (NI), respiratory intervention (RI), psychological intervention (PSI), and integrated physical intervention (IPI), on functional status, quality of life, muscle strength, pulmonary function, and safety in patients with amyotrophic lateral sclerosis (ALS). We searched nine databases, PubMed, Cochrane, Embase, Scopus, Web of Science, CNKI, CBM, WFPD, and CSTJ, for randomized controlled trials of ALS patients. The primary outcome was the Amyotrophic Lateral Sclerosis Functional Rating Scale-Revised (ALSFRS-R) score. Secondary outcomes were the McGill Quality of Life Questionnaire (McGill-QoL), Medical Research Council (MRC)-sum score, Forced Vital Capacity (FVC), and Fatigue Severity Scale (FSS) score. This NMA was conducted using random-effect models to calculate the standard mean difference (SMD) and 95% confidence interval (CI). All types of supplemental interventions had some benefit for patients with ALS. EI had a beneficial effect on the ALSFRS-R score (SMD: 1.01; 95% CI 0.50–1.51), FVC (SMD: 0.78; 95% CI 0.02–1.55), McGill-QoL (SMD: 0.71 95% CI 0.33–1.08), and MRC (SMD: 1.11; 95% CI 0.08–2.14). RI had a beneficial effect on the ALSFRS-R score (SMD: 0.83 95% CI 0.12–1.55). IPI had a beneficial effect on the ALSFRS-R score (SMD: 0.65 95% CI 0.06–1.24). NI had a beneficial effect on the McGill-QoL (SMD: 0.63 95% CI 0.02–1.23). The current study findings support a multimodal intervention strategy with an emphasis on EI for slowing disease progression in patients with ALS.

## Introduction

Amyotrophic lateral sclerosis (ALS) is a fatal neurodegenerative disease that affects the front part of the spinal cord, motor nerve nuclei in the brainstem, the pyramidal tract, and motor neurons in the upper and lower limbs^1^. ALS results in muscle weakness, myasthenia gravis, myofascial fibrillation, medullary palsy, pyramidal signs, dyspnea, weight loss, and cardiac arrhythmia^2,3^. Respiratory failure, malnutrition, and aspiration pneumonia are the leading causes of death in ALS patients^4^.

ALS has remained an incurable disease^5^. After being diagnosed with ALS, patients should seek multidisciplinary care teams to control their symptoms, according to the most recent ENFS and EALSC working group guidelines^6^. Three ALS medications, Riluzole, Edaravone and Relyvrio, can be prescribed to slow disease progression and increase life expectancy^7–9^. However, Edaravone has been linked to serious side effects such as bruising, headaches, dermatitis, and gait disturbances^10^, while *Riluzone* is associated with increased liver enzymes, vomiting, weakness, and stomach upset^11^. *Relyvrio* has several adverse effects, including diarrhea, stomach pain, nausea, cold symptoms, upper respiratory tract infection, tiredness, drooling a lot, and dizziness^12^.

Along with medications, ALS patients can choose non-pharmacological interventions such as respiratory intervention, psychological, integrated physical intervention, nutritional intervention, and exercise intervention. All of these can help patients improve their functional ability, stabilize their mood, improve their quality of life, and slow the progression of ALS pathology^13–15^. To the best of our knowledge, however, no previous studies compared the efficacy of the five interventions on the clinical symptoms of ALS patients.

Although choosing a safe and effective intervention is an important factor to consider as part of a multidisciplinary care plan, the relative efficacy of the plans is unclear^16,17^. As a result, healthcare providers have struggled to find the optimal treatment plan for their patients. The purpose of this study was to compare the efficacy of supplemental interventions for ALS patients.

## Materials and methods

### Program and registration

This NMA was conducted under the guidelines on preferred reporting items for systematic reviews and meta-analysis (PRISMA). The protocol for this systematic review and network meta-analysis has been registered within PROSPERO (CRD42023459965).

### Inclusion and exclusion criteria

Inclusion criteria were: (1) 18 years or older with definite or probable ALS as determined by the EI Escorial criteria or its revised version^18,19^; (2) respiratory treatment (RI) that included respiratory muscle training and inspiratory muscle training; psychological treatment (PSI) that included meditation training and non-meditative mindfulness intervention; integrated physical intervention (IPI) that included repetitive transcranial magnetic stimulation; or massage, acupuncture, and pharyngeal electrical stimulation; nutritional treatment (NI) that included supplementation of branched-chain amino acids, L-threonine, vitamin E, creatine, CoQ10, milk whey proteins, acetyl‐L‐carnitine, hypercaloric enteral nutrition, and high-calorie foods; exercise treatment (EI) that included muscle exercise, resistance exercise, a monitored exercise program, aerobic exercise, endurance exercise, and a combination of aerobic exercise and strength program; control (CON) that included receiving standard care, usual care, no treatment, sham expiratory training, sham inspiratory training, sham repetitive transcranial magnetic stimulation, continuous care, placebo, or usual stretching; (3) outcomes included any of the following measurements: Amyotrophic Lateral Sclerosis Functional Rating Scale Revised (ALSFRS-R) score, McGill quality of life questionnaire (McGill-QoL), and 36-item Short Form Survey (SF-36). Secondary outcomes included Forced Vital Capacity (FVC), Maximal Inspiratory Pressure (MIP), Medical Research Council (MRC)-sum score, Manual Muscle Testing (MMT), Maximum Voluntary Isometric Contraction (MVIC), Lower-Extremity Muscle Strength, Fatigue Severity Scale (FSS) score, and Visual Analogy Scale for Fatigue (VAS-F) score; and (4) the study design of a randomized controlled trial. Exclusion criteria were conference abstracts, reviews, syntheses, letters, guidelines, non-randomized controlled trials, studies with incomplete data, and non-English publications.

### Search strategies

Two independent researchers searched nine databases: PubMed, Cochrane, Embase, Scopus, Web of Science, CNKI, CBM, WFPD, and CSTJ, from inception to June 2023. We conducted the online search for the articles published between the periods using the following logic: (1) "amyotrophic lateral sclerosis" or "ALS"; (2) "respiratory treatment," "psychology treatment," "respiratory treatment," "psychology treatment," "physiotherapy," "nutritional treatment," and "exercise treatment"; and (3) "randomized controlled trial" or "RCT" or "randomized" or "placebo.” The search strategy was developed first for the PubMed database and then adapted for the other sources. Experts in ALS and intervention then reviewed these sources to make sure they were complete and accurate. The detailed search strategy is presented in Supplementary Appendix 1.

### Study selection

The Endnote 21 software was used to select eligible articles, exclude duplicates, and filter out articles that did not meet the inclusion criteria. Two researchers independently read the titles and abstracts, initially selected potential studies, screened the full texts, and chose the studies that ultimately met the requirements. A third investigator resolved any discrepancies, if necessary. Finally, eligible studies were documented and recorded using a PRISMA flow chart.

### Data extraction

Two independent investigators extracted the data. A third investigator was called in to help resolve data discrepancies and provide a consensus for decision-making. The data were then recorded by author and year, number of participants (male and female ratio), age, treatments, and outcome index.

### Risk-of-*bias* assessment

The Cochrane risk-of-bias assessment tool (Review Manager 5.4) was used to evaluate selection bias, implementation bias, measurement bias, follow-up bias, reporting bias, and other potential biases. The quality of studies was graded by two evaluators and classified as “low, high, or unclear risk of bias.” If a disagreement arose, a third evaluator stepped in to resolve the issue. The risk of bias is presented in Supplementary Appendix 2.

### Statistical analyses

NMA was performed using STATA 14.0 MP software to incorporate an indirect comparison of non-pharmacological interventions for ALS. Because the data in this NMA are all continuous variables with different scales for each outcome indicator, the standard mean difference (SMD) and 95% confidence interval (CI) were calculated. A *p*-value ≤ 0.05 was considered statistically significant for between-group differences. Consistency and inconsistency tests between studies were used to improve study stability. A funnel plot was used to detect publication bias. The effectiveness of the interventions was ranked using the surface under the cumulative ranking curve (SUCRA) and mean rank, which considers all possible rankings and uncertainties in intervention effects; a greater SUCRA value indicated a more effective intervention.

## Results

### Literature search and study inclusion

**Figure 1:**
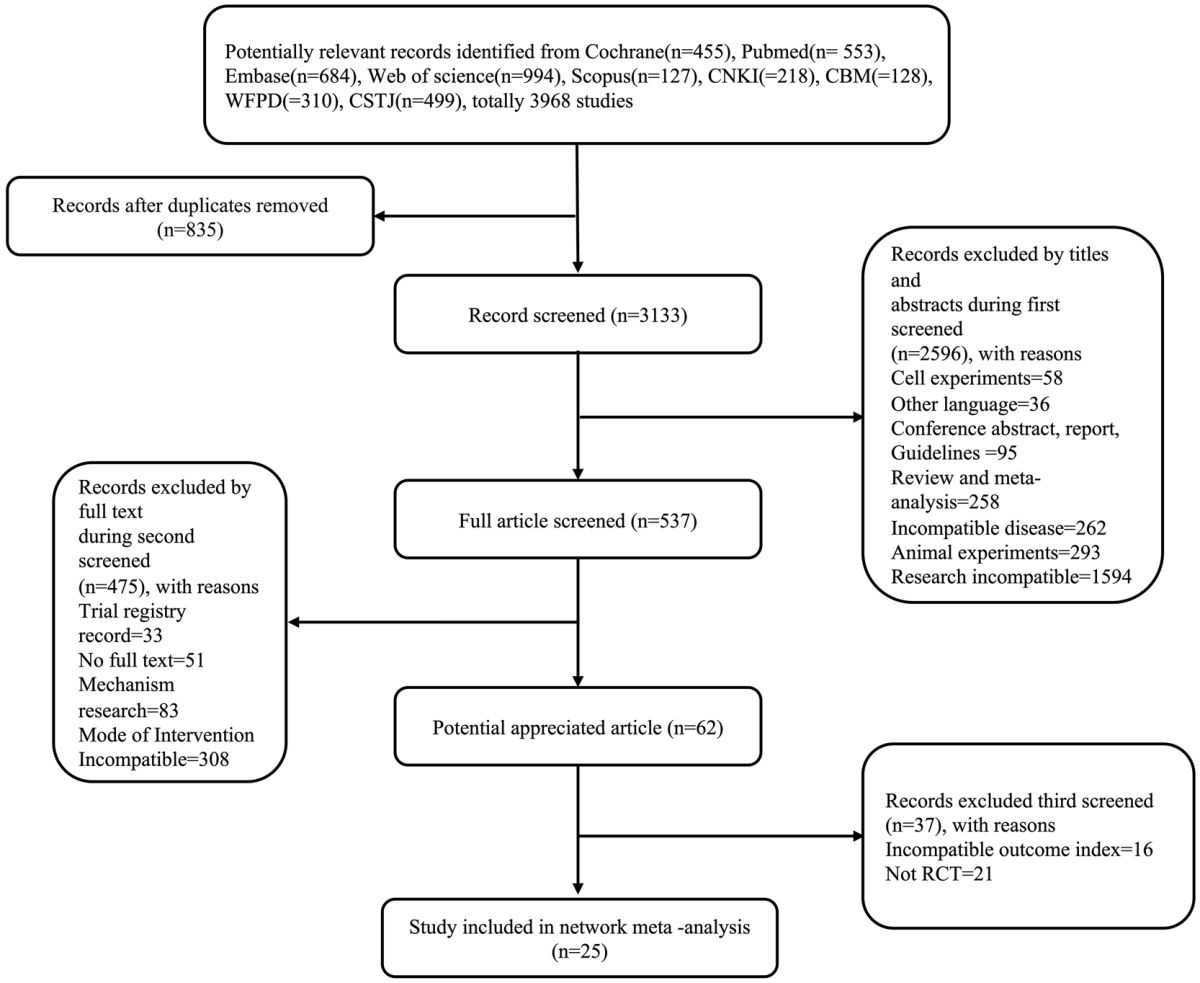
Flow chart showing literature selections.

As shown in Fig. 1, the initial research on the nine databases yielded 3,968 studies. After 835 duplicates were removed, the first round of screening resulted in the selection of 537 studies based on the titles and abstracts. After reading the full articles, 62 studies were chosen. Thirty-seven of these were subsequently excluded due to an incompatible outcome index (*n* = 16) or not being an RCT (*n* = 21). The remaining 25 eligible studies were included in the NMA.

### General characteristics

Table 1 summarizes the 25 RCTs that included 1226 patients eligible for data analysis. The studies included four respiratory interventions, two psychological interventions, six physiotherapy interventions, six nutritional interventions, and seven exercise interventions conducted between 1996 and 2022 and were published in English.

**Table 1:** Characteristics of studies used for analysis.

Studies	Interventions	N	Age, mean (SD)	Gender (M/F)	Baseline ALSFRS-R	Outcomes	Duration (week)
Cheah et al. 2009	RI	9	54.2 (9.8)	6/3	38.2 (6.5)	FVC	12
	CON	10	53.4 (9.5)	6/4	38.9 (2.7)		
Pinto et al. 2012	RI	13	57.14 (9.3)	7/6	34.39 (3.64)	ALSFRS, FVC	32
	CON	13	56.8 (8.7)	11/2	33.5 (3.8)		
Plowman et al. 2019	RI	24	63.1 (10)	17/7	36.6 (6.3)	ALSFRS-R, FVC	8
	CON	24	60.1 (10.3)	12/12	37.5 (6.1)		
Plowman et al. 2023	RI	23	62.0 (9.6)	15/8	39.7 (5.7)	ALSFRS-R, FVC	12
	CON	22	64.5 (11.4)	13/9	37.5 (5.2)		
Pagnini et al. 2016	PSI	50	57.93 (11.33)	31/19	29.83(4.83)	ALSSQoL-R	8
	CON	50	63.41 (10.16)	33/17	32.17(6.64)		
Pagnini et al. 2022	PSI	19	63.9 (8.3)	9/10	39.63(9.21)	ALSSQoL-R	5
	CON	19	62.3 (8.6)	10/9	37.24(6.82)		
Di Lazzaro et al. 2006	PHI	7	60.6 (13)	5/2	40.8 (5.5)	ALSFRS- R, MMT	24
	CON	8	65.7 (7.2)	3/5	37.5 (7.3)		
Zanette et al., 2008	PHI	5	59.4 (9.2)	4/1	36.0 (3.4)	SF-36, FSS	2
	CON	5	60.2 (8.7)	3/2	34.4 (2.3)		
Di Lazzaro et al. 2009	PHI	10	60.2 (6.7)	8/2	32 (7.2)	ALSFRS-R, MMT	48
	CON	10	55.1 (14.0)	7/3	31.3 (6.9)		
	Con	15	53.33 (9.52)	9/6	25. 91 (5. 54)		
Ai qun son et al. 2021	PHI	30	53 (7)	16/14	24.67 (3.69)	ALSFRS-R, FVC	12
	CON	30	54 (8)	17/13	26.10 (3.60)		
Ju peng et al. 2019	PHI	15	53.17 (9.14)	10/5	25. 77 (5. 82)	ALSFRS-R, MRC	12
	CON	15	53.33 (9.52)	9/6	25. 91 (5. 54)		
Herrmann et al. 2022	PHI	10	76.0 (6.41)	5/5	31.5 (7.92)	ALSFRS- R	3 days
	CON	10	57.5 (14.07)	3/7	36.0(10.51)		
Tandan et al. 1996	NI	31	59.6 (10.6)	53/42	NA	FVC, MRC	24
	NI	32					
	CON	32					
Desnuelle et al. 2001	NI	144	62.5 (11.2)	79/65	NA	FVC, MMT, VAS fatigue	48
	CON	144	65.7 (9.4)	79/65			
Kaufmann et al. 2009	NI	75	56.5 (10.8)	40/35	35.3 (5.5)	ALSFRS-R, FVC, FSS	36
	CON	75	57.4 (11)	46/29	35.6 (5.0)		
de Carvalho Silva et al. 2010	NI	8	53 (13.33)	14/2	29.1 (2.4)	ALSFRS- R	16
	CON	8			27.0 (3.8)		
Beghi et al. 2013	NI	42	40–70	24/18	43 (2.22)	ALSFRS- R, FVC, McGill-QoL, MRC	48
	CON	40		26/14	42 (12.59)		
Wills et al. 2014	NI	9	57.5 (15.4)	4/5	25.5 (9·4)	ALSFRS-R, FVC	16
	NI	8	64.0 (6.9)	3/5	30.7 (7.0)		
	CON	7	63.2 (9.4)	3/4	23.0 (3.5)		
Drory et al. 2001	EI	14	58 (13.2)	8/6	29.35 (3.77)	ALSFRS- R, SF-36, MMT, FSS	48
	CON	11	60.7 (16.4)	6/5	26.23 (4.3)		
Lunetta et al. 2016	EI	30	61.1 (10.1)	21/9	39.1 (4.7)	ALSFRS- R, FVC, McGill-QoL	24
	CON	30	60.3 (9.9)	17/13	38.3 (5.1)		
Braga et al. 2018	EI	24	60.5 (16.30)	18/6	42.92 (3.51)	ALSFRS- R	24
	CON	24	63.0 (6.85)	14/10	41.13 (4.83)		
Merico et al. 2018	EI	23	61.6 (10.6)	10/13	36.1 (4.71)	ALSFRS- R, MRC, FSS	5
	CON	15	59.8 (14.7)	4/10	34.5 (3.6)		
Ferri et al. 2019	EI	8	50.7 (3.3)	2/6	40.4 (1.5)	ALSFRS- R, ALSSQoL, Lower-extremity strength	12
	CON	8	55.5 (5.95)	2/6	35 (3.4)		
van Groenestijn et al. 2019	EI	27	60.9 (10.0)	19/8	42.3 (3.5)	ALSFRS- R, FVC, Lower-extremity strength	16
	CON	30	59.9 (10.7)	22/8	42.3 (4.2)		
Kalron et al. 2021	ET	14	58.5 (13.2)	9/5	35.7 (5.3)	ALSFRS- R, FVC, SF-36	12
	CON	14	60.4 (14.7)	6/8	37.5 (5.6)		

*RI* respiratory intervention, *PSI* psychological intervention, *PHI* physiotherapy intervention, *NI* nutritional intervention, *EI* exercise intervention, *ALSFRS-R* Amyotrophic Lateral Sclerosis Functional Rating Scale-Revised, *FVC* Forced Vital Capacity, *ALSSQOL-R* Amyotrophic Lateral Sclerosis-Specific Quality of Life, *MMT* Manual Muscle Testing, *SF-36* Short Form (36), *FSS* Fatigue Severity scale, *McGill-QoL* McGill Quality of Life Questionnaire, *MRC* Medical Research Council, *VAS fatigue* Visual Analog Scale for Fatigue, *NA* Not Available.

### Quality assessment

The randomization methods used in the 25 RCTs were random sequence generation (*n* = 14), random number table method (*n* = 3), computer-generated randomization lists (*n* = 3), permuted block randomization (*n* = 4), and duplo-cego randomization (*n* = 1). Except for EI and PSI, allocation concealment was used to prevent selection bias in all remaining RCTs. For participant and personnel blinding, 11 studies used double blinding, two studies used single blinding, and the remaining studies did not specify. For detection bias, eight studies were assessor-blinded, while the remaining studies did not report. One study had a selective bias. Our study was free of attrition and other biases. Overall, the quality assessment of the included studies was high.

### Small sample effect

**Figure 2:**
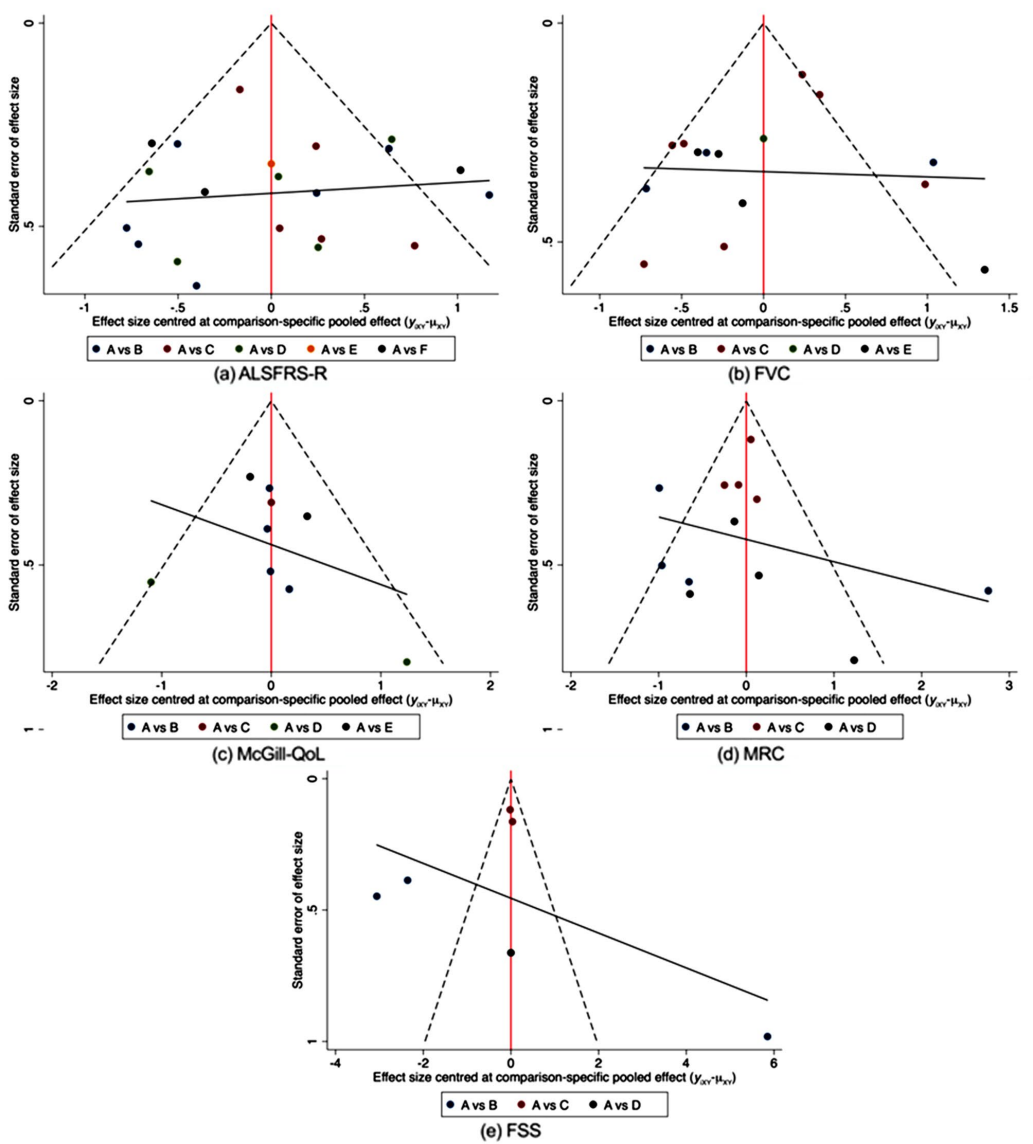
Funnel plot. *ALSFRS-R* Amyotrophic lateral sclerosis Functional Rating Scale-Revised, *FVC* Forced Vital Capacity, *McGill-QoL* McGill Quality of Life Questionnaire, *MRC* Medical Research Council, *FSS* Fatigue Severity Scale.

Publication bias is a critical factor in determining the validity and generalization of conclusions in systemic reviews and meta-analyses. In this meta-analysis, a funnel plot was used to detect publication bias, with effect size on the horizontal coordinate and standard error on the vertical coordinate. As the sample size increases and the standard error decreases, trials are likely to converge around the true underlying effect size. The funnel plots of the trials used in the current study indicate a lower likelihood of publication bias in the ALSFRS-R score (Fig. 2a), FVC (Fig. 2b), McGill-QoL (Fig. 2c), MRC (Fig. 2d), and FSS score (Fig. 2e).

### Consistency and inconsistency analysis

An inconsistency analysis of our network meta-analysis indicates a stable model with no significant evidence of discrepancy in indirect comparisons. The restricted maximum likelihood method was used in conjunction with a variance–covariance matrix proportional to 0.5I (3) + 0.5 J (3,3,1). Furthermore, the specific test for inconsistency within the model yielded a chi-square value and probability value, which are shown in Supplementary Appendix 3, supporting our conclusion that the observed data do not deviate significantly from what would be expected under a consistent model. This analysis provides strong evidence to support the consistency assumption in our network meta-analysis.

### Eligible comparison network plots

**Figure 3:**
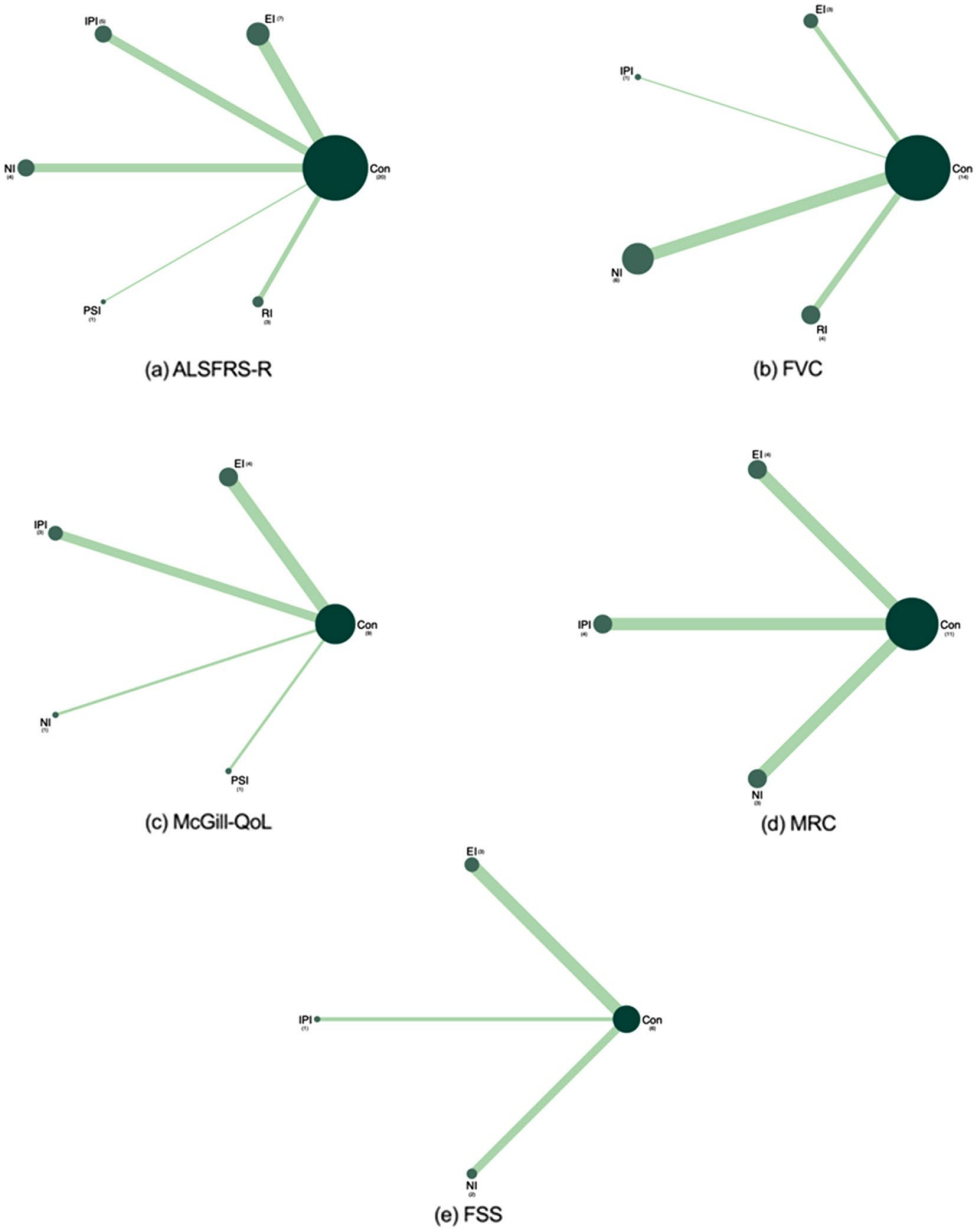
Network of eligible comparisons for all interventions included in the analysis. *ALSFRS-R* Amyotrophic lateral sclerosis Functional Rating Scale-Revised, *FVC* Forced Vital Capacity, *McGill-QoL* McGill Quality of Life Questionnaire, *MRC* Medical Research Council, *FSS* Fatigue Severity Scale. The size of the nodes is proportional to the number of participants assigned to the intervention and the thickness of the lines is proportional to the number of randomized trials that studied the respective direct comparison.

Figure 3a depicts the network diagram of eligible comparisons for the primary outcome of the ALSFRS-R scores. Figure 3b–e show the network diagrams of eligible comparisons for the secondary outcomes of FVC, McGill-QoL, MRC, and FSS scores. Five nodes represented five different interventions, and controls served as a bridge for indirect comparison and resulted in five pairs of comparisons.

### Primary outcomes

Table 2 depicts the SUCRA rankings of the five interventions on ALSFRS-R score. EI had the best SUCRA (84.5%) on functional capacity, followed by RI (70.9%), IPI (56.8%), NI (43%), and PSI (37.3%).

**Table 2:** SUCRA rankings from six-node analysis.

Parameters	Rank	Intervention	SUCRA (%)
ALSFRS-R	1	Exercise intervention	84.5
	2	Respiratory treatment	70.9
	3	Physiotherapy	56.8
	4	Nutritional intervention	43.0
	5	Psychological treatment	37.3
	6	Control	7.5
FVC	1	Exercise intervention	81.7
	2	Physiotherapy	65.9
	3	Respiratory treatment	64.4
	4	Control	26.5
	5	Nutritional treatment	11.5
McGill-QoL	1	Exercise intervention	85.8
	2	Nutritional treatment	76.9
	3	Psychological treatment	40.7
	4	Physiotherapy	33.3
	5	Control	13.3
MRC	1	Exercise intervention	89.9
	2	Physiotherapy	60.5
	3	Nutritional treatment	26.4
	4	Control	23.2
FSS	1	Physiotherapy	61.4
	2	Control	56.5
	3	Nutritional treatment	55.9
	4	Exercise intervention	26.3

*ALSFRS-R* Amyotrophic lateral sclerosis Functional Rating Scale-Revised, *FVC* Forced Vital Capacity, *McGill-QoL* McGill Quality of life Questionnaire, *MRC* Medical Research Council, *FSS* Fatigue Severity Scale, *SUCRA* surface under the cumulative ranking curve.

**Figure 4:**
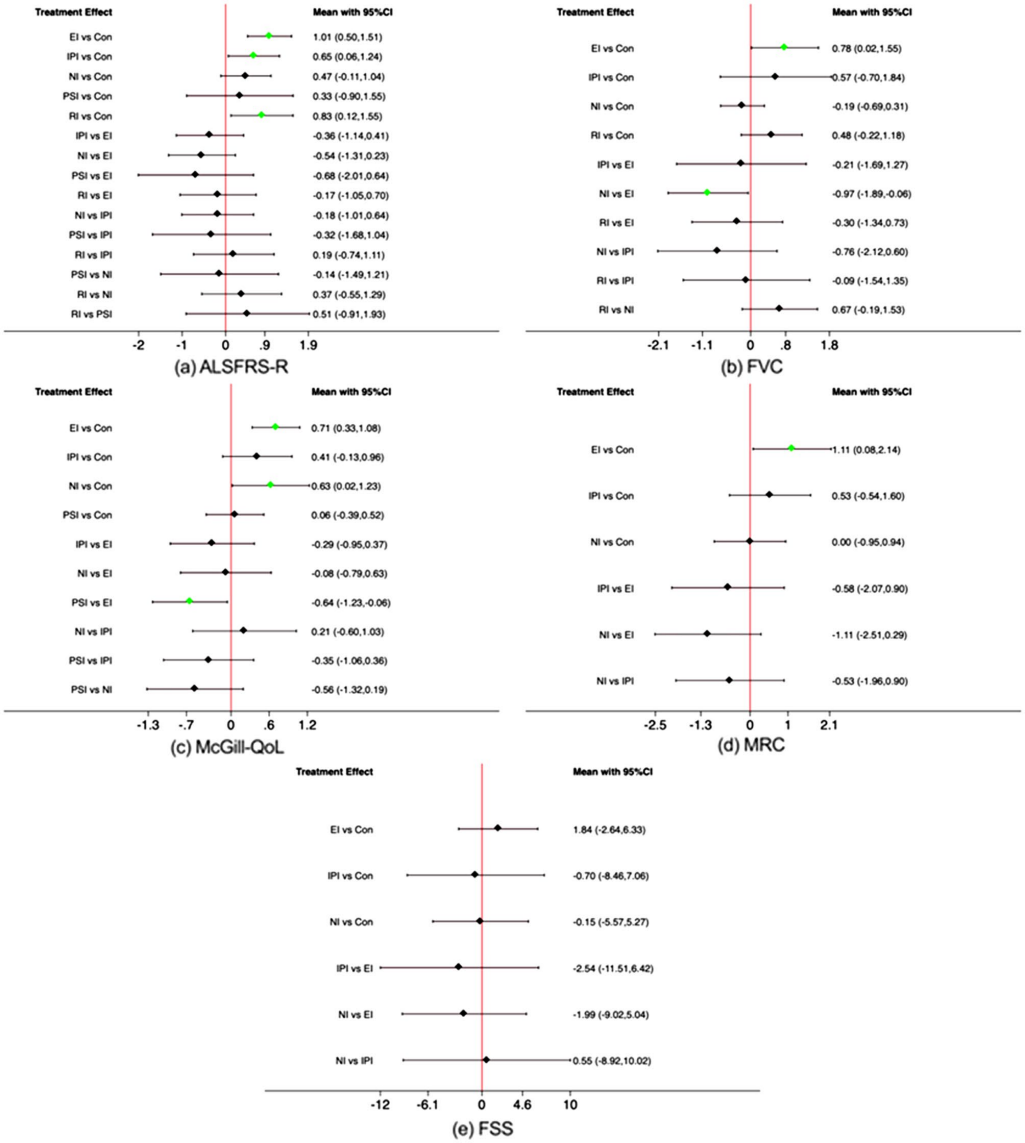
Forest Plot for eligible comparisons for all interventions included in the analysis. *ALSFRS-R* Amyotrophic lateral sclerosis Functional Rating Scale-Revised, *FVC* Forced Vital Capacity, *McGill-QoL* McGill Quality of Life Questionnaire, *MRC* Medical Research Council, *FSS* Fatigue Severity Scale.

Figure 4a depicts a forest plot of the ALSFRS-R score. Compared to Con, the effect on functional capacity was greater in EI (SMD = 1.01, 95% CI 0.50 ~ 1.51), RI (SMD = 0.83, 95% CI 0.12 ~ 1.55), IPI (SMD = 0.65, 95% CI 0.06 ~ 1.24), NI (SMD = 0.47, 95 CI − 0.11 ~ 1.04), and PSI (SMD = 0.33, 95 CI − 0.90 ~ 1.55). Taken together, the primary outcomes of the NMA indicate that EI is the most effective strategy for slowing the functional capacity decline due to ALS.

### Secondary outcomes

Table 2 depicts the SUCRA rankings of the five interventions on the secondary outcomes. On FVC, EI had the highest SUCRA (81.7%), followed by IPI (65.9%), RI (64.6%), and NI (11.4%). On the McGill-QoL scale, EI had the highest SUCRA (85.8%), followed by NI (76.9%), PSI (40.7%), and IPI (33.3%). On MRC, EI had the highest SUCRA (89.9%), followed by IPI (60.5%) and NI (26.4%). On the FSS score, IPI had the highest SUCRA (61.4%), followed by CON (56.5%), NI (55.9%), and EI (26.3%).

Figure 4 represents the forest plots of the five different interventions on the secondary outcomes. Compared to CON, the effect on FVC was greater in EI (SMD = 0.78, 95% CI 0.02 ~ 1.55), IPI (SMD = 0.57, 95% CI − 0.70 ~ 1.84), RI (SMD = 0.48, 95% CI − 0.22 ~ 1.18), and NI (SMD =  − 0.19, 95% CI − 0.69 ~ 0.31). Furthermore, EI had a greater effect on FVC (SMD =  − 0.97, 95% CI − 1.89 ~  − 0.06) than NI. Compared to Con, the effect on McGill-QoL was greater in EI (SMD = 0.71, 95% CI 0.33 ~ 1.08) and NI (SMD = 0.63, 95% CI 0.02 ~ 1.23), while the effect on MRC was greater in EI (SMD = 1.11, 95 CI 0.08 ~ 2.14) and IPI (SMD = 0.53, 95 CI − 0.54 ~ 1.60). Compared to CON, none of the five interventions had a significant effect on the FSS score. Taken together, the outcomes of the study suggest that EI is the most effective intervention strategy for the secondary outcomes of ALS patients.

## Discussion

This study examined the efficacy of five interventions on functional status, pulmonary function, muscle strength, quality of life, and the severity of fatigue by performing a systematic review and network meta-analysis of 25 RCTs involving 1,226 ALS patients. To the best of our knowledge, this is the first study to show that, despite intervention-specific benefits, EI outperformed RI, IPI, NI, and PSI on all metrics except severity of fatigue.

In agreement with the current study findings, the beneficial effect of EI on the functional status of ALS patients has been reported in many previous studies. For example, unsupervised structured home exercise or progressive muscle training combined with aerobic exercise resulted in improved functional status in terms of the ALSFRS-R score, Functional Independence Measure (FIM), and Barthel scores, as well as improved quality of life in ALS patients^20–22^. The beneficial effects of EI are also reported in animal studies. For instance, swimming training modifies muscle metabolism by increasing muscle bioenergetics and decreasing oxidative stress. This training also slows grip strength loss and increases lifespan in the G93A-SOD1 mouse, which expresses a G93A mutant form of human copper-zinc superoxide dismutase 1 (SOD1) and can be used for ALS research^23^.

There are some explanations for the suppressive effects of EI on disease progression in ALS patients. First, exercise training activates satellite cells that reside between the basal lamina and sarcolemma of adult skeletal myofibers. These cells aid in muscle repair and regeneration in ALS patients. Second, exercise training increases the production of neuroprotective hormones^24,25^. Resistance training and aerobic exercise, for example, have been shown to increase growth hormone secretion^26–30^, which promotes growth hormone (GH) binding to intracellular receptors on the cell membrane and stimulates target cell proliferation^31^.

Because respiratory failure is unavoidable and a leading cause of death in ALS patients, RI has become a focal point for the disease. For example, ALS rodent models lose a lot of intercostal motor neurons and phrenic neurons. This makes the respiratory system much harder to use by placing more metabolic stress on the remaining neurons^32,33^. This type of unfavorable compensatory mechanism hastens motor neuron death^34^. In contrast, RI has been shown to improve brain-derived neurotrophic factor (BDNF)-dependent synthesis of phrenic neuronal output rates, which improves ALS patients' quality of life, ability to perform actions, and ability to breathe^35,36^. One systematic review demonstrated that RI improves respiratory muscle function with an effect size of 1.48 and a 12-month mean survival extension^37^.

IPI has also demonstrated unique therapeutic benefits on the ALSFRS-R scale score. Scalp acupuncture, which is widely used in research and clinical settings for neurodegenerative diseases, has shown promise as a treatment for ALS. For example, eight-week acupuncture therapy helped a 55-year-old female ALS patient by improving dorsiflexion strength and easing cramps, fasciculations, and lower limb pain^38^. Four-week acupuncture injection point therapy improved speech clarity, motor function, and muscle strength in two ALS patients^39^. Additionally, massage has been shown to help relieve clinical symptoms of ALS, such as muscle stiffness and cramping, by activating the parasympathetic nervous system through force on the skin surface and exerting pressure on the muscles to increase blood flow^40^.

Because oxidative stress is an important hypothesis for ALS pathogenesis, antioxidant research has become popular^41^. Vitamins, particularly vitamin B12 and vitamin E, play a protective role in the development of ALS^42^. Creatine monohydrate administration has a positive trend in the survival of ALS patients with no adverse events^43^. Malnutrition and weight loss are common in ALS patients^44,45^. High-calorie and ultra-high-calorie supplements can play an important role in increasing the body weight of ALS patients^46^.

PSI may help ALS patients with depression and improve their quality of life. For example, hypnosis and mindfulness-based psychological interventions improved patients' quality of life and relieved their pain, depression, and anxiety while also slowing disease progression^47,48^. Physiologically, PSI has been shown to increase telomere length and activity in leukocytes, promote telomeric DNA synthesis, and counteract the telomere depletion seen in the pathology of ALS^49–51^, while suppressing oxidative stress through increased levels of ROS-degrading enzymes^52^.

Fatigue is a common symptom seen at all stages of ALS pathology and is associated with worse functioning, poor quality of life, increased pain intensity and disease severity, and muscle weakness. Female ALS patients are more often affected by fatigue^53^. None of the non-pharmacological interventions included in the current study have shown a clear improvement in the FSS score compared to CON.

Even though it outperforms other interventions, selecting or recommending EI with the highest SUCRA ranking should not be a routine procedure for patients with ASL due to the following reasons. First, the efficacy of EI may vary depending on the stage of ALS^54,55^, potentially leading to bias in interpreting the intervention's results. That is, patients in the early stages of ALS are more likely to maintain the physical ability required for participation in such interventions, excluding those in later stages, for whom exercise may be impractical or, at the very least, significantly more difficult. Second, the potential bias in exercise modalities and durations adds another layer of complexity^16,56^, implying that the therapeutic benefits observed in this study may not be uniformly attributable to exercise itself, but rather to its specific characteristics, such as intensity, frequency, and patients' physical condition at the start of the intervention. This nuance highlights the possibility of varying response rates across the ALS population, complicating the task of generalizing our findings to the larger ALS community. To address these concerns, future research should take a more stratified methodological approach, ensuring that participants are not only representative of the broader ALS spectrum, but also categorized according to disease stage, physical capacity, and intervention specifics. Such stratification would enable a more granular analysis of the data, facilitating a nuanced understanding of the relationship between exercise interventions and ALS progression. Taken together, there may be insufficient evidence to draw a firm conclusion about the superior efficacy of EI for ALS patients, raising the risk of misinterpreting the efficacy hierarchy of the interventions in slowing the disease progression.

This meta-analysis included RCTs with varying degrees of blinding, ranging from double-blind to studies with unspecified blinding procedures^57–81^. We included studies regardless of blinding statutes due to a scarcity of ALS intervention research and methodological considerations. As a result, heterogeneity in blinding may introduce bias, compromising the internal validity of the results. Blinding, particularly double blinding, is the gold standard for RCTs, but its practical application in ALS trials presents significant challenges. They include the difficulty of masking certain interventions, ethical concerns about placebo control for life-altering conditions, and the possibility of unblinding due to the intervention's obvious effects^57,59,61^. Transparency in reporting the blinding process, including successes and failures, will allow for a more nuanced interpretation of trial results. It is critical to understand that developing blinding methods in ALS trials is a methodological and ethical requirement for ensuring the credibility of research findings and maintaining patient care integrity^82,83^.

Finally, the ALSFRS-R is a validated tool for tracking the progression of disability in the bulbar, motor, and respiratory domains in ALS^84^. Thus, individual subscale scores of the ALRFRS-R rather than total scores may provide more useful information about the progression of disability in ALS^85,86^, as the effects of the interventions included in the current study may be domain-specific. However, due to limited access to original datasets, our study relied on a total score rather than individual subscale scores. As a result, the therapeutic effects of the interventions on ALSFRS-R scores observed in the current study should be interpreted with caution and more specific in terms of bulbar, motor, and respiratory domains. This represents a limitation in our analysis framework and necessitates a thorough discussion. As a result, it is critical to recognize that, while the ALSFRS-R is a useful tool for tracking disease progression and patient function, using a total score rather than individual subscale scores may introduce bias when assessing the effectiveness of specific interventions that it directly queries. By contrast, a recent study found that the ALSFRS-R bulbar subscale had poor to fair screening accuracy for detecting global pharyngeal dysphagia, while the ALSFRS-R swallowing item had poor overall screening accuracy for both global swallowing and safety status^87^. As a result, future research could benefit from using metrics that assess respiratory function independently of the ALSFRS-R. This results in a more accurate representation of the efficacy of interventions, lowering bias risks and increasing the clinical relevance of the findings.

## Conclusions

To the best of our knowledge, we are the first to compare the effectiveness of the five non-pharmacologic interventions for ALS patients. We conducted a systematic review and NMA of 25 RCTs with a total of 1226 patients and showed that EI is more effective than the other interventions, but some benefits are unique to each intervention. The current study findings support a multimodal intervention strategy with an emphasis on EI for slowing disease progression in patients with ALS. At the same time, it is also critical to interpret these findings with the knowledge that the stage of disease may influence the feasibility and effectiveness of these interventions. Further research is required to determine the full scope of these interventions' applicability and to optimize intervention strategies for diverse patient profiles across the ALS spectrum.

### Supplementary Information

Supplementary Information.

Supplementary Figure 1.

Supplementary Table 1.

Supplementary Legends.

## Data Availability

The datasets analyzed and generated during the current study are available from the corresponding author on reasonable request.
