# Supplementary figures and images for: Efficacy of non-pharmacological interventions for individuals with amyotrophic lateral sclerosis: systematic review and network meta-analysis of randomized control trials
Source: Sci Rep. 2024 May 18;14:11365. doi: 10.1038/s41598-024-62213-w (PMC11102473; doi:10.1038/s41598-024-62213-w)

**
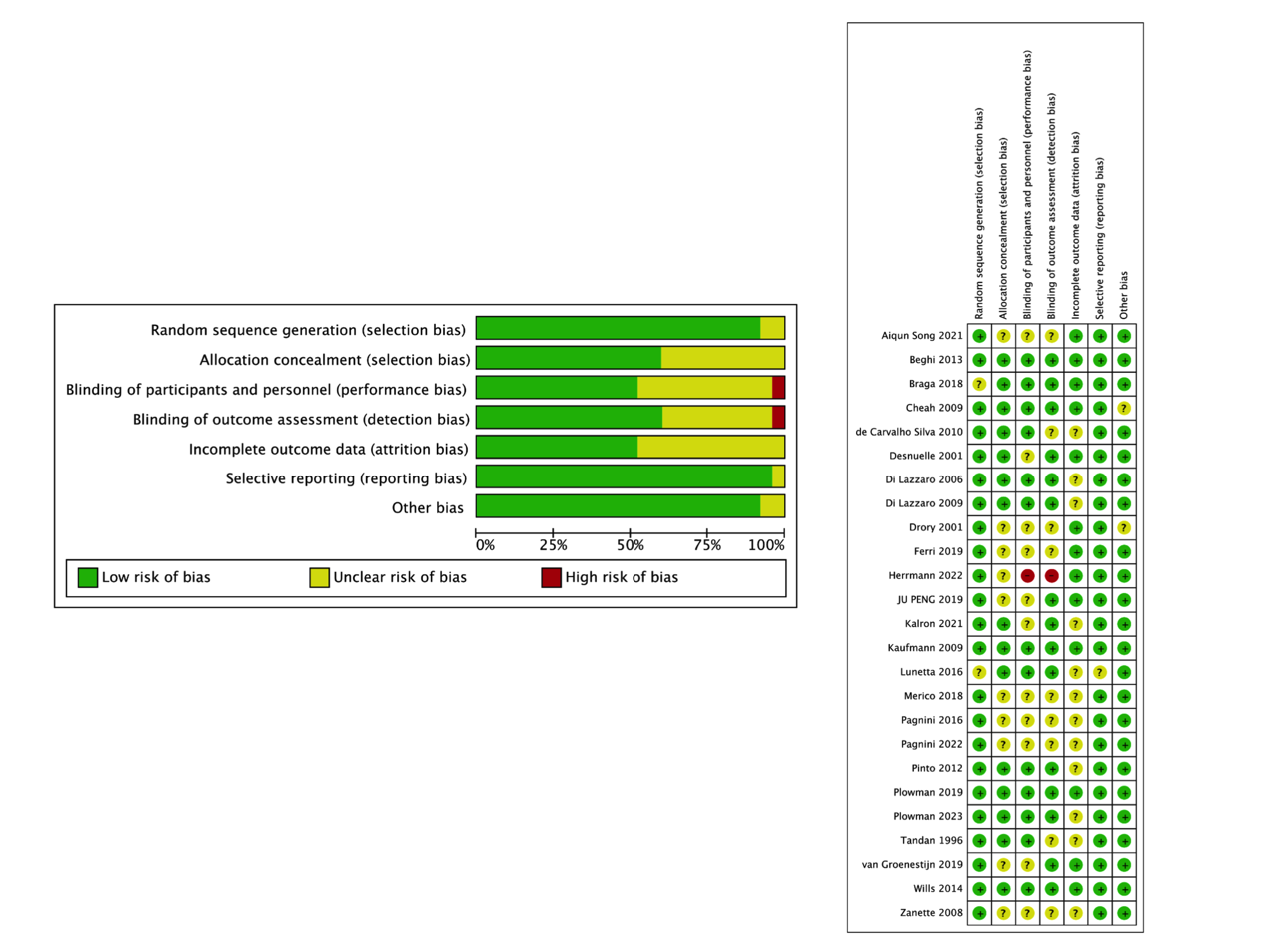
**

**Supplementary figure 1**. Risk-of-bias assessment.

Supplement: Supplementary file 2 — Supplementary Figure 1. [file 41598_2024_62213_MOESM2_ESM.docx]
