# Supplementary material for: Efficacy of non-pharmacological interventions for individuals with amyotrophic lateral sclerosis: systematic review and network meta-analysis of randomized control trials
Source: Sci Rep. 2024 May 18;14:11365. doi: 10.1038/s41598-024-62213-w (PMC11102473; doi:10.1038/s41598-024-62213-w)
Supplement: Supplementary file 3 — Supplementary Table 1. [file 41598_2024_62213_MOESM3_ESM.docx]

| Supplementary Table 1. Results of inconsistence analysis. | | | |
| --- | --- | --- | --- |
| Parameters | chi2 | P value |  |
| ALSFRS-R | 0.11 | 0.7450 |  |
| FVC | 0.31 | 0.5765 |  |
| McGill-QoL | 0.34 | 0.5588 |  |
| MRC | 0.12 | 0.7289 |  |
| FSS | 0.03 | 0.8599 |  |
| ALSFRS-R: Amyotrophic lateral sclerosis Functional Rating Scale-Revised, (b) FVC: Forced Vital Capacity, (c) McGill-QoL: McGill Quality of life Questionnaire, (d) MRC: Medical Research Council, (e) FSS: Fatigue Severity Scale. | | | |
