# Supplementary material for: Efficacy of non-pharmacological interventions for individuals with amyotrophic lateral sclerosis: systematic review and network meta-analysis of randomized control trials
Source: Sci Rep. 2024 May 18;14:11365. doi: 10.1038/s41598-024-62213-w (PMC11102473; doi:10.1038/s41598-024-62213-w)
Supplement: Supplementary file 4 — Supplementary Legends. [file 41598_2024_62213_MOESM4_ESM.docx]

Supplementary Appendix 1. PubMed Searching history.
